# Inhibition of HDAC6 activity protects dopaminergic neurons from alpha-synuclein toxicity

**DOI:** 10.1038/s41598-020-62678-5

**Published:** 2020-04-08

**Authors:** Laetitia Francelle, Tiago F. Outeiro, Gudrun A. Rappold

**Affiliations:** 1 Department of Human Molecular Genetics, Institute of Human Genetics, Ruprecht-Karls-University Heidelberg, Heidelberg, Germany; 2 Department of Experimental Neurodegeneration, Center for Biostructural Imaging of Neurodegeneration, University Medical Center Goettingen, Goettingen, Germany; 3 Max Planck Institute for Experimental Medicine, Goettingen, Germany

**Keywords:** Regeneration and repair in the nervous system, Parkinson's disease, Molecular medicine

## Abstract

The neuropathological hallmarks of Parkinson’s disease include preferential vulnerability of dopaminergic neurons of the substantia nigra pars compacta, and accumulation of intraneuronal protein inclusions known as Lewy bodies. These inclusions contain, among other proteins, aggregated alpha-synuclein and histone deacetylase 6 (HDAC6). In our study we found that selective inhibition of HDAC6 activity by Tubastatin A has protective effects in a rat model of Parkinson’s disease. We provide evidence that this protection may be due to the activation of chaperone-mediated autophagy through the up-regulation of key members of this pathway. Moreover, Tubastatin A significantly inhibited the expression of a toxic form of alpha-synuclein that is phosphorylated at serine position 129. Tubastatin A treatment also permitted to partially modulate neuroinflammation. Taken together, our study highlights the neuroprotective effects of Tubastatin A in a rat model of Parkinson’s disease and provides mechanistic insight in Tubastatin A-mediated protection against alpha-synuclein toxicity and substantia nigra degeneration. These findings are of potential therapeutic value in Parkinson’s disease and other synucleinopathies.

## Introduction

Parkinson’s disease (PD) is a neurodegenerative disorder characterized by intracellular inclusions of aggregated and misfolded proteins, such as alpha-synuclein. Altered alpha-synuclein expression and accumulation of alpha-synuclein aggregates in cells can lead to alpha-synucleinopathies. In PD patients, dopamine-secreting neurons of the substantia nigra pars compacta show selective degeneration^1^. The selective vulnerability of the substantia nigra may be explained by factors enhancing alpha-synuclein toxicity^2,3^. The identification of cell-autonomous factors modulating alpha-synuclein toxicity may constitute possible targets for therapeutic intervention in synucleinopathies^4^. We hypothesized that posttranslational modifications of alpha-synuclein might be one of these factors. Indeed, ubiquitination, sumoylation, phosphorylation, truncation, oxidation, acetylation and glycation regulate the physiological functions of alpha-synuclein *in vitro* and *in vivo*^5,6^. Some of these modifications promote pathological processes by inducing alpha-synuclein conformational changes and affecting its oligomerization^5,7^. For example, we found that deacetylation of alpha-synuclein at lysine positions 6 and 10 by the deacetylase sirtuin 2 (SIRT2) affects its aggregation and toxicity^8^. Therefore, lysine acetylation might be a key regulatory mechanism governing alpha-synuclein structure, function, aggregation and toxicity. Lysine acetylation can be modulated by histone acetyltransferases (HATs) and histone deacetylases (HDACs). HDAC6 is considered a promising therapeutic target for neurodegenerative diseases since it deacetylates non-histone substrates, such as alpha-tubulin, heat-shock protein 90 (Hsp90), and peroxiredoxins I and II, and regulates microtubule-based transport, cell motility, endocytosis, cell migration, autophagy and aggresome formation^9–11^. The role of HDAC6 has been previously addressed in models of PD^12–14^. Recent studies addressing the role of the ubiquitin-proteasome system in a mouse model of PD suggest that HDAC6 could mediate the dissociation of Hsp90-containing heat shock factor 1 (Hsf1) complex, and protect dopaminergic neurons from cytotoxic alpha-synuclein aggregates by stimulating the formation of aggresome-like inclusions^14^. However, these findings still have to be further validated.

The goal of the present study was to improve our understanding of the molecular mechanisms that are impaired in PD, and to verify the potential neuroprotective effects of an increased level of alpha-synuclein acetylation by the inhibition of HDAC6. For this, we used an established rat model of PD-like neurodegeneration, induced by human alpha-synuclein overexpression in the substantia nigra pars compacta^15^. We already used this rat model to test the effect of other putative therapeutic targets, such as *sirtuin 2* (SIRT2)^8^. We adapted the experimental design in order to phenocopy early-onset PD defects. To specifically inhibit HDAC6, we used Tubastatin A and tested it as a preventive drug in this early onset model of PD. We found that inhibition of HDAC6 activity by Tubastatin A has a disease modifier effect in the substantia nigra pars compacta by protecting dopaminergic neurons against alpha-synuclein toxicity, accompanied by reduced astrogliosis. Our results demonstrate that Tubastatin A treatment lead to differential protein expression compared to the vehicle. Interestingly, we found that Hsc70 and Lamp2 proteins were upregulated by Tubastatin A, whereas alpha-synuclein was downregulated. Importantly, we did not detect phosphorylation of alpha-synuclein at serine position 129, an accepted marker of pathological alpha-synuclein, in the rats treated with Tubastatin A. Overall, our work supports the hypothesis that specific inhibition of HDAC6 activity increases alpha-synuclein acetylation, up-regulates key members of the chaperone-mediated autophagy, and reduces alpha-synuclein expression and toxicity. Therefore, HDAC6 is a potential therapeutic target for PD and other alpha-synucleinopathies.

## Results

### Tubastatin A protects dopaminergic neurons in the rat substantia nigra against alpha-synuclein toxicity

**Figure 1:**
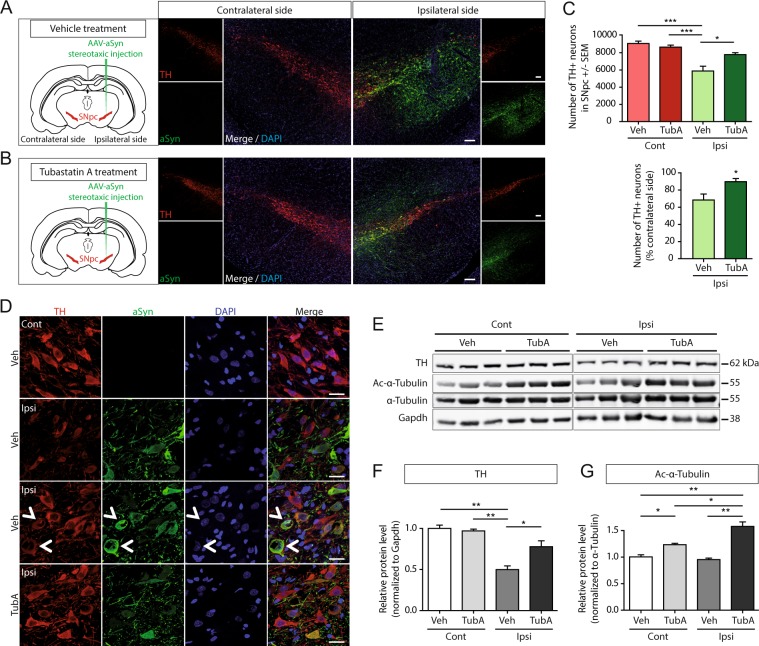
Tubastatin A protects dopaminergic neurons from alpha-synuclein toxicity in rats. (**A**,**B**, left panels) Schematic of the experimental paradigm used to overexpress alpha-synuclein in rat substantia nigra (SN) by unilateral stereotaxic injection of AAV2-human alpha-synuclein (ipsilateral side). Two days post-stereotaxic injection, the rats received a daily intraperitoneal injection of vehicle (Veh) (**A**) or Tubastatin A (TubA) (**B**) for 14 days. Two weeks after infection, brains were processed for histological evaluation by immunostainings or for biochemical analysis. (**A**,**B**, right panels) Representative coronal sections of rat brain displaying human alpha-synuclein overexpression (green, ipsilateral side) on the tyrosine hydroxylase (TH)-positive dopaminergic neurons of the substantia nigra (red). Scale bar = 100 μm. (**C**) Stereological quantification of TH-positive neurons in the contralateral (Cont) and ipsilateral (Ipsi) sides of the substantia nigra of all animals (*P < 0.05; ***P < 0.001; n = 7 sections through substantia nigra/5–6 rats/group) (top) and percentage of nigral TH-positive neurons expressed as the mean % of the contralateral side in the human alpha-synuclein-injected animals at 2 weeks post-injection treated with vehicle or Tubastatin A (*P < 0.05; n = 7 sections through substantia nigra/5–6 rats/group) (bottom). (**D**) High-magnification representative immunofluorescence confocal microscopy images showing the co-localization of TH (red) and human alpha-synuclein (green) in the substantia nigra of alpha-synuclein-injected animals at 2 weeks post-injection, treated with vehicle (top three rows) or Tubastatin A (bottom row), on the contralateral (first row) or ipsilateral (three last rows) sides. Scale bar = 20 μm. White arrows point on abnormal shaped neurons. (**E**) Representative immunoblots for TH, acetylated-alpha-tubulin (Ac-α-tubulin), total-alpha-tubulin (α-tubulin) and Gapdh, and (**F**,**G**) quantification of TH normalized with Gapdh, and acetylated alpha-tubulin (Ac-α-tubulin) normalized with total alpha tubulin (α-tubulin), expressed as relative protein level, in the contralateral and ipsilateral sides of alpha-synuclein-injected rats treated with vehicle or Tubastatin A (*P < 0.05; **P < 0.01; n = 6–7/group). kDa, kilodalton.

The purpose of the experimental design was to model the early onset of PD and to test Tubastatin A as a preventive drug. To investigate the putative effect of Tubastatin A on the toxicity of alpha-synuclein *in vivo*, we used an established rat model of PD^15^. Briefly, we injected adeno-associated virus (AAV) serotype 2, driving the expression of human alpha-synuclein, in the substantia nigra pars compacta of rats by stereotaxic injection (Fig. 1A,B, left panels). For two weeks, starting two days after the stereotaxic injection, animals were daily treated with either vehicle (0.5% DMSO, PBS, 2% Tween 20) (Fig. 1A) or with Tubastatin A at a low concentration (15 mg/kg) (Fig. 1B) by intraperitoneal injection. At the end of the treatment, animals were sacrificed and we assessed the integrity of dopaminergic neurons using tyrosine hydroxylase (TH) immunohistochemical staining (Fig. 1A,B, right panels, red staining). In the injected hemisphere, expression of human alpha-synuclein was detected using an antibody specific for human alpha-synuclein (Fig. 1A,B, right panel, ipsilateral side, green staining). We quantified the TH-positive cells in the substantia nigra of the alpha-synuclein injected (ipsilateral) and non-injected (contralateral) sides from the alpha-synuclein-injected rats treated with either vehicle or Tubastatin A (Fig. 1C). Two-way ANOVA confirmed no difference between contralateral sides between the two groups of rats. However, in the ipsilateral side of vehicle-treated rats, human alpha-synuclein expression induced loss of the TH expressing neurons. In the substantia nigra of Tubastatin A-treated rats, the reduction in TH signal was significantly less pronounced than in the substantia nigra of the vehicle-treated animals. The number of TH-positive neurons was not significantly different in the Tubastatin A-treated group compared to the contralateral side of both groups (Fig. 1C). Moreover, cells strongly expressing alpha-synuclein in the vehicle group exhibited reduced TH immunoreactivity (Fig. 1D, second and third lines), and some of these cells showed an abnormal shape (Fig. 1D, third line, white arrows), suggesting alpha-synuclein toxicity in TH-positive cells. We also observed a lower expression of human alpha-synuclein in the Tubastatin A-treated rats compared to vehicle (Fig. 1A,B).

Quantification of the TH expression level by Western blot analysis supported the protective effect of Tubastatin A (Fig. 1E,F). The TH protein expression was significantly lower in the alpha-synuclein-injected side of vehicle-treated rats compared with the other groups, whereas it was significantly higher in the alpha-synuclein-injected side of Tubastatin A-treated rats compared to the vehicle-treated rats, and not different to the non-injected side of both groups.

Incidentally, the concentration of injected Tubastatin A was effective in inducing a significant increase of alpha-tubulin acetylation (Fig. 1G). The levels of acetylated alpha-tubulin were higher in the ipsilateral side of Tubastatin A-treated rats substantia nigra compared to the contralateral side, suggesting that inhibition of HDAC6 deacetylation activity is even stronger in the presence of high levels of alpha-synuclein (Fig. 1G).

### Effects of tubastatin A treatment on protein expression levels

**Figure 2:**
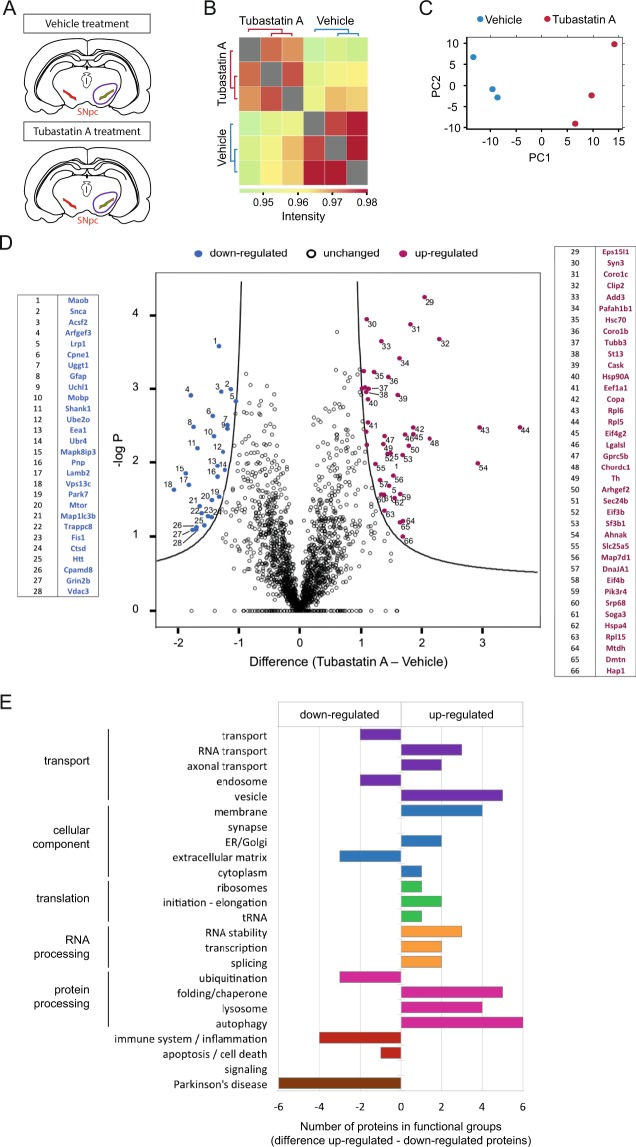
Proteomic analysis of vehicle- and Tubastatin A-treated alpha-synuclein-injected rats. (**A**) Brain region including substantia nigra (purple circle) of three vehicle-treated (aSyn + Veh) and three Tubastatin A-treated (aSyn + TubA) alpha-synuclein-injected rats were used to perform label-free quantitative proteomic analysis. (**B**) Hierarchical clustering heatmap of Pearson correlation coefficients between all animals used in this experiment. The highest correlations are obtained between animals within the same group (vehicle or Tubastatin A). **(C)** Principle component analysis (blue: vehicle; red: Tubastatin A). **(D)** Volcano plot of differentially regulated proteins in response to Tubastatin A treatment in alpha-synuclein injected rats. Proteins were graphed by fold change (difference) and significance (-Log p) using a false discovery rate of 0.01 and an SO of 0.1. Significantly up-regulated proteins were shown as red dots and down-regulated proteins were shown as blue dots using the Perseus software. (**E**) Each down- or up-regulated protein from the Tubastatin A group was assigned a unique label that best represented its function. The number of proteins within a specific function is shown.

To better understand the mechanism underlying the protective effects of Tubastatin A and to investigate its effect on protein expression levels, we employed a label-free quantitative mass spectrometry approach. The substantia nigra of alpha-synuclein-injected rats was analyzed. Three vehicle-treated rats and three Tubastatin A-treated rats were used for this experiment (Fig. 2A). Rats from the same group showed a similar differential protein expression (Fig. 2B,C). Using Perseus software, we generated a volcano plot that revealed significantly down- and up-regulated proteins. The expression levels of sixty-six proteins were found to be significantly altered between vehicle and Tubastatin A-treated animals, using a fold change cutoff of 1 (Fig. 2D). The protein expression of alpha-synuclein (Snca) was found to be down-regulated (−1.8 FC). Specific protein changes induced by Tubastatin A treatment included proteins responsible for transport, ubiquitination, inflammation and PD. After attributing a functional tag to each of the proteins, we found that the Tubastatin A group included a larger number of up-regulated proteins that play a role in vesicle transport, folding/chaperoning, lysosome activity and autophagy (Fig. 2E).

### Tubastatin A increases the protein expression levels of Hsc70 and Lamp2A in presence of alpha-synuclein

To further investigate the molecular mechanisms underlying the protective effect of Tubastatin A against dopaminergic neuronal loss associated with alpha-synuclein expression, we hypothesized - based on data from the proteomics analysis - that Tubastatin A might affect the protein expression levels of human alpha-synuclein through the activation of the autophagy pathway.

**Figure 3:**
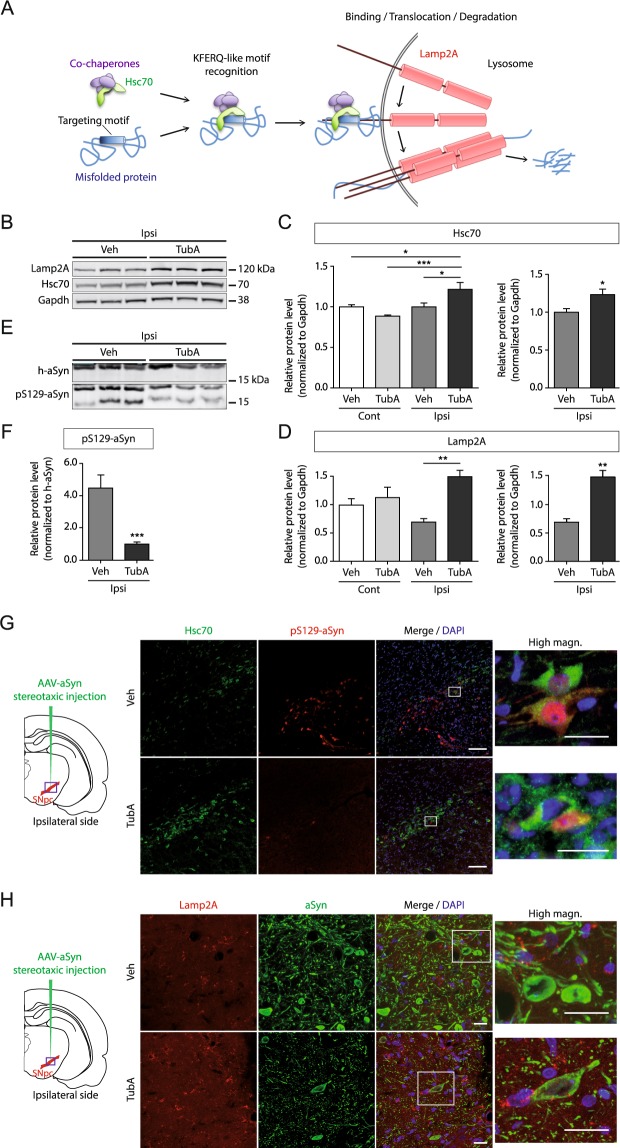
Tubastatin A treatment up-regulates chaperone-mediated autophagy members and reduces phosphorylation of alpha-synuclein at serine 129. (**A**) Schematic of the chaperone-mediated autophagy. Proteins degraded by the chaperone-mediated autophagy are identified in the cytosol by Hsc70, the constitutive member of the cytosolic family of chaperones of 70 kDa. Hsc70 and co-chaperones bind to the targeting KFERQ-like motif in the substrate protein and bring it to the surface of lysosomes. Binding of the substrate to the cytosolic tail of the receptor protein Lamp2A promotes Lamp2A multimerization to form a translocation complex. Upon unfolding, substrate proteins cross the lysosomal membrane and reach the lysosomal matrix where they undergo complete degradation. (**B**) Representative immunoblots for Hsc70, Lamp2A and Gapdh in the ipsilateral (Ipsi) side of alpha-synuclein-injected rats, and (**C**,**D**) quantification of Hsc70 and Lamp2A normalized with Gapdh, expressed as relative protein level, in the contralateral (Cont) and ipsilateral sides of alpha-synuclein-injected rats treated with vehicle (Veh) or Tubastatin A (TubA) (*P < 0.05; **P < 0.01; ***P < 0.001; n = 6–7/group). (**E**) Representative immunoblots for human alpha-synuclein (h-aSyn) and phosphorylated alpha-synuclein at serine position 129 (pS129-aSyn), and (**F**) quantification of pS129-aSyn normalized with h-aSyn, expressed as relative protein level, in the ipsilateral side of alpha-synuclein-injected rats treated with Veh or TubA (***P < 0.001; n = 6–7/group). (**G**,**H**) Representative immunofluorescence confocal microscopy images showing the double staining of Hsc70 (green) and pS129-aSyn (red) (**G**), or the double-staining of Lamp2A (red) and human alpha-synuclein (green) (**H**), in the substantia nigra of alpha-synuclein-injected animals (purple square on brain representation) treated with vehicle (top) or Tubastatin A (bottom) on the ipsilateral side. Scale bar = low magnification, 100 µm; high magnification, 20 μm.

Autophagy is a dynamic process that degrades damaged organelles, misfolded proteins and aggregates, and is typically impaired in neurodegenerative diseases^16^. Different steps in the autophagic pathway are disrupted in different models of alpha-synuclein toxicity, including cargo loading in chaperone-mediated autophagy^17,18^, autophagosome fusion with lysosomes^19^, and axonal transport^20^. Given the function of HDAC6, we focused on autophagic pathways that degrade soluble misfolded proteins, such as chaperone-mediated autophagy (Fig. 3A). Alpha-synuclein is a substrate of the chaperone-mediated autophagy and contains a KFERQ-like motif, which is VKKDQ at position 95^17^. The Hsc70 protein can bind to a misfolded protein by its KFERQ peptide motif and bring it to the lysosomal membrane, where Lamp2A is recruited and multimerized to translocate the misfolded protein into the lysosome for degradation. We determined protein levels of two key proteins involved in chaperone-mediated autophagy, Hsc70 and Lamp2A, by Western blot analysis. Specifically, we analyzed Hsc70 and Lamp2A in the substantia nigra of Tubastatin A-treated or control animals expressing alpha-synuclein. We found both proteins to be significantly increased in the alpha-synuclein-injected substantia nigra of the Tubastatin A-treated rats, but not of the vehicle-treated animals (Fig. 3B–D), suggesting that Tubastatin A may lead to the activation of the chaperone-mediated autophagy pathway in the presence of human alpha-synuclein.

### Tubastatin A reduces phosphorylation of alpha-synuclein at serine 129

In Lewy bodies of PD patients, the predominant pathological post-translational modification of alpha-synuclein is phosphorylation at serine position 129 (pS129-alpha-synuclein)^21^. We detected this toxic form of phosphorylated alpha-synuclein in the alpha-synuclein-injected substantia nigra (Fig. 3E,G). Two bands corresponding to the phosphorylated alpha-synuclein were detected by Western blot. Even if the predicted size of alpha-synuclein is 14 kDa, the full-length and phosphorylated forms migrated higher on gels, at around 18 kDa (Fig. 3E). We quantified the band at 18 kDa (Fig. 3F). The other band around 14 kDa might be a truncated form, as found in Lewy Bodies^21^, and the C-terminal truncated alpha-synuclein, containing the S129 region, has been associated to increased toxicity in the rat model overexpressing full-length alpha-synuclein in the substantia nigra^22^. Quantification of protein levels by Western blot analysis showed that pS129-alpha-synuclein was significantly reduced in the Tubastatin A-treated rats compared to the vehicle-treated ones (Fig. 3F). Immunostainings showed that fewer cells were positive for pS129-alpha-synuclein in the Tubastatin A group compared to the vehicle group (Fig. 3G). Furthermore, we observed an inverse relationship between Hsc70 and pS129-alpha-synuclein (Fig. 3G), and between Lamp2A and human alpha-synuclein (Fig. 3H). The pS129-alpha-synuclein positive cells showed lower levels of Hsc70 protein expression in the vehicle group, whereas more Hsc70 and less pS129-alpha-synuclein positive cells were observed in the Tubastatin A group at the alpha-synuclein injection area (Fig. 3G). Immunostainings also showed that Lamp2A signal was stronger in the Tubastatin A group (Fig. 3H). Taken together, human alpha-synuclein expression in neurons is lower in the Tubastatin A group compared with the vehicle group (Fig. 3H), which is correlated with the weak pS129-alpha-synuclein staining in these neurons (Fig. 3G).

### Influence of tubastatin A on alpha-synuclein acetylation

We have recently shown that SIRT2 deacetylates alpha-synuclein on lysine positions 6 and 10^8^. Since HDAC6 interacts with alpha-synuclein^12–14^, we hypothesized that HDAC6 could also deacetylate alpha-synuclein. Thus, we next investigated whether Tubastatin A also affected the acetylation of alpha-synuclein.

**Figure 4:**
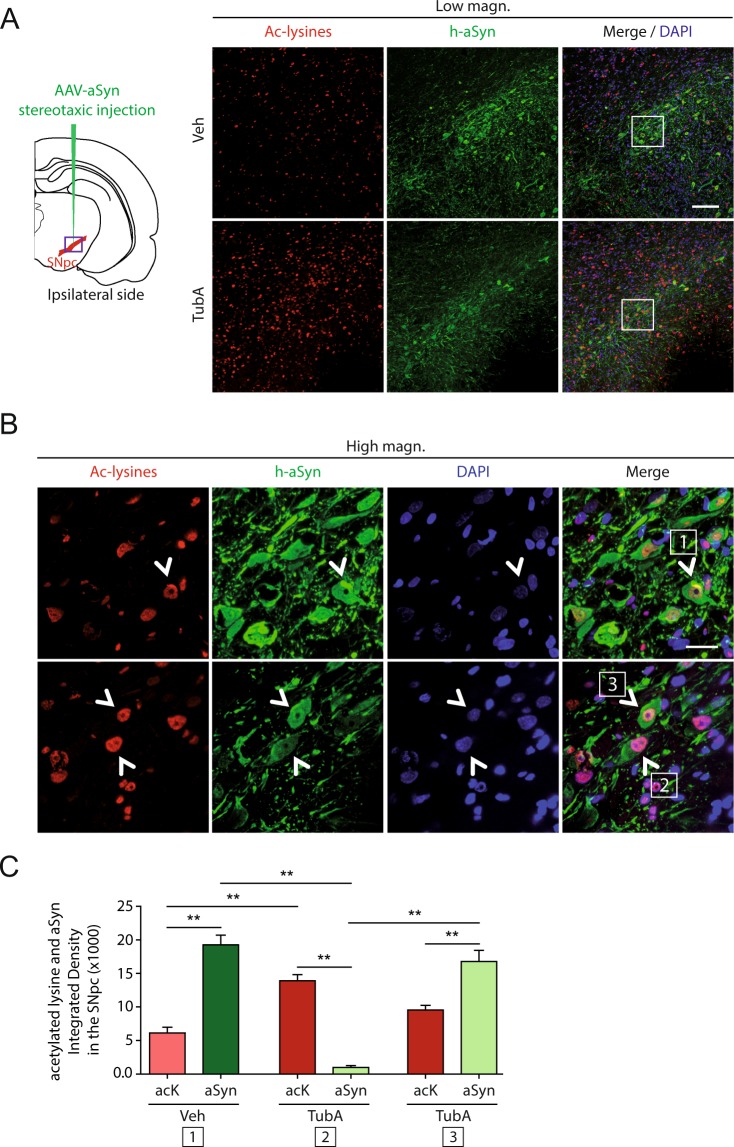
Effect of tubastatin A on the acetylation of alpha-synuclein. (**A**,**B**) Representative immunofluorescence confocal microscopy images of the substantia nigra area (purple square on brain representation) showing staining for acetylated lysines (Ac-lysines) (red), human alpha-synuclein (h-aSyn) (green) and DAPI (blue) at low magnification (**A**) and at high magnification (**B**), and quantification of fluorescence using integrated density of acetylated lysines (acK)- and alpha-synuclein–positive cells (**P < 0.01; n = 20 cells/rat/group in all rats) (**C**) in the alpha-synuclein-injected side of rats treated with vehicle (Veh) or Tubastatin A (TubA). White squared numbers (**B**) show representative co-stained cells used for quantification. (**C**) Scale bar = low magnification, 100 µm; high magnification, 20 μm. White squares on the top panel show the localization of the high magnification area. White arrows point on co-localized double staining cells.

Using immunostainings, we found that Tubastatin A treatment increased the overall levels of acetylated lysines, compared with the vehicle-treated group (Fig. 4A,B, resp. low and high magnification). We then quantified the fluorescence intensity of the cells that were positive for both acetylated lysines and human alpha-synuclein (Fig. 4C). In the vehicle group, alpha-synuclein staining was stronger compared to the acetylated lysine staining in the majority of tested neurons (Fig. 4B, high magnification and Fig. 4C; “**1”**). In the Tubastatin A group, two groups of cells were observed, presenting either a stronger staining for acetylated lysines (Fig. 4B, high magnification and Fig. 4C; **“2”**) or, similar to the vehicle group, a stronger staining for alpha-synuclein (Fig. 4B, high magnification and Fig. 4C; **“3”**). This indicates that under our conditions Tubastatin A seems to partially increase lysine acetylation, suggesting that a higher acetylation level of alpha-synuclein might lead to the reduction of its expression, or to the increase of its degradation.

### Tubastatin A reduces astrocyte reactivity in the substantia nigra of alpha-synuclein-injected rats

**Figure 5:**
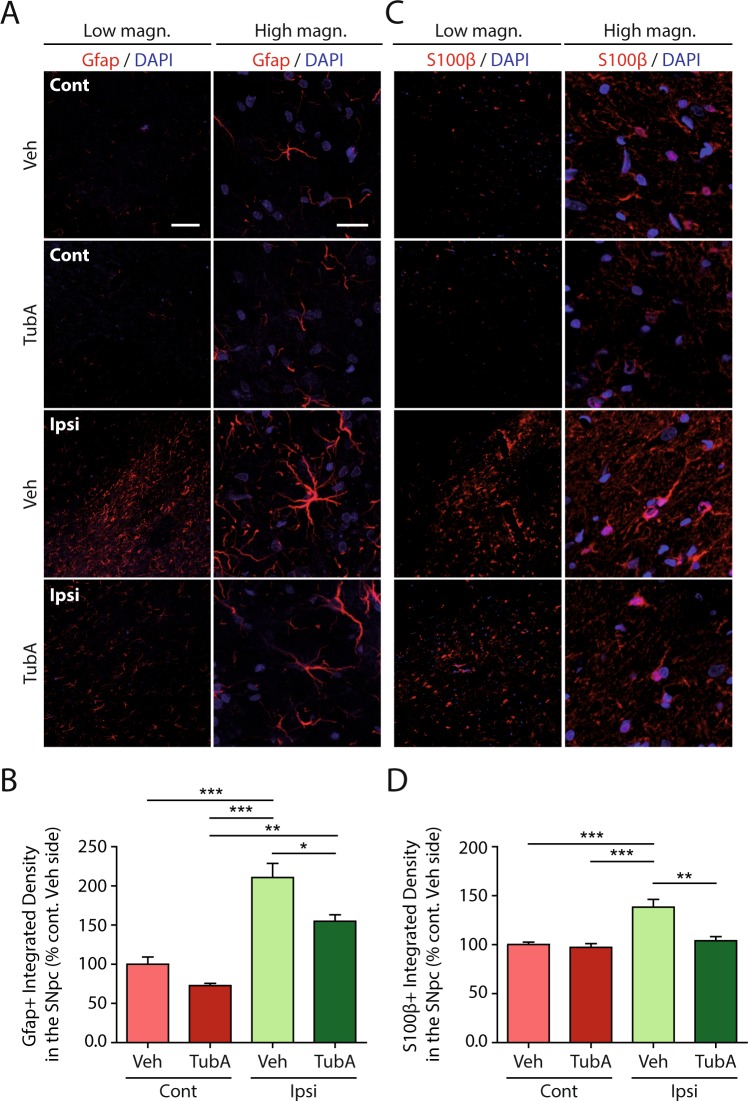
Tubastatin A reduces astrocyte reactivity in the substantia nigra of alpha-synuclein-injected rats. (**A**,**C**) Representative immunofluorescence confocal microscopy images of the substantia nigra area showing staining for Gfap (**A**), S100β (**C**) and DAPI in the contralateral (Cont) and ipsilateral (Ipsi) sides of alpha-synuclein-injected rats treated with vehicle (Veh) or Tubastatin A (TubA). Scale bar = low magnification, 100 µm; high magnification, 20 μm. (**B**,**D**) Quantification of fluorescence using integrated density of Gfap (**B**), S100β (**D**) –positive fluorescence staining intensity area in the contralateral and ipsilateral sides of the substantia nigra, expressed as percentage of the contralateral side of the alpha-synuclein-injected and vehicle-treated rats (% Cont Veh side) (*P < 0.05; **P < 0.01; ***P < 0.001; n = 7/group).

Our proteomics data suggested that inflammation might be modulated by Tubastatin A treatment. Therefore, we next investigated a possible link between alpha-synuclein, HDAC6 inhibition, chaperone-mediated autophagy activation and neuroinflammation. The antioxidant transcription factor Nrf2 has previously been shown to protect cells against human mutated A53T-alpha-synuclein toxicity through the autophagy-lysosome pathway in astrocytes, mediating alpha-synuclein degradation^23^. In neurodegenerative diseases glial cells undergo morphological and functional changes referred to as reactivity^24,25^. Therefore, we analyzed different astrocytic markers, such as Gfap and S100β. We observed a higher number of astrocytes stained with Gfap and S100β in the alpha-synuclein-injected rats treated with the vehicle (Fig. 5A,C, third row, low magnification; 5B,D) when compared with the control groups (Fig. 5A,C, first and second rows; 5B,D).

Moreover, these cells became hypertrophic, a sign of increased reactivity (Fig. 5A,C, third row, high magnification). Interestingly, in the alpha-synuclein-injected substantia nigra of the Tubastatin A-treated rats, the integrated density of Gfap and S100β-positive cells was lower compared with the alpha-synuclein-injected substantia nigra of the vehicle group (Fig. 5A,C, fourth row, low magnification; 5B,D), and their morphology was intermediate between the controls and the vehicle-treated alpha-synuclein-injected group (Fig. 5A,C, fourth row, high magnification). Therefore, astrocyte reactivity induced by human alpha-synuclein could be significantly reduced by Tubastatin A, possibly promoting the survival of nigral dopaminergic neurons.

## Discussion

**Figure 6:**
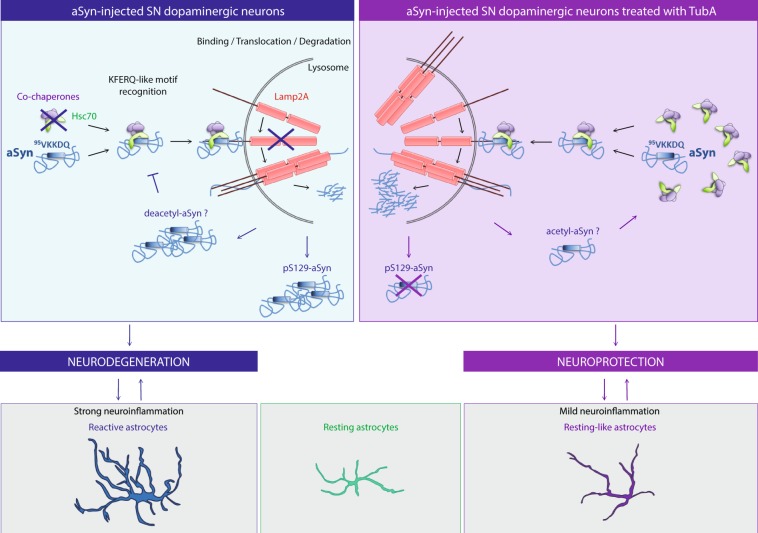
Proposed mechanisms of neuroprotective effects of Tubastatin A against alpha-synuclein toxicity. The left side of the figure shows the potential mechanisms leading to dopaminergic neuron loss in the alpha-synuclein-injected substantia nigra (SN). On the right side of the figure, the effects of Tubastatin A are represented. Under these conditions, astrocytes are in a resting-like state, resulting in milder neuroinflammation, which may promote neuronal survival.

Our study demonstrated that specific inhibition of HDAC6 activity by Tubastatin A protects rat dopaminergic neurons in substantia nigra pars compacta against alpha-synuclein toxicity. Our quantitative proteomic analysis revealed that Tubastatin A treatment modulated pathways known to be altered in PD. We showed that human alpha-synuclein expression was reduced in Tubastatin A-treated rats compared with vehicle-treated rats. The decrease in alpha-synuclein expression could be explained by the conformational change of alpha-synuclein induced by its higher acetylation in presence of Tubastatin A, which would facilitate its uptake and degradation by key members of the chaperone mediated autophagy, as schematized in Fig. 6. In the substantia nigra of alpha-synuclein-injected rats, Hsc70 interacts with alpha-synuclein and pS129-alpha-synuclein through the alpha-synuclein KFERQ-like motif, which brings it to the Lamp2A membrane receptor at the lysosome surface to be degraded. As human alpha-synuclein and pathological pS129-alpha-synuclein accumulate over time^26^, it may impair chaperone-mediated autophagy, causing further alpha-synuclein accumulation. This hypothesis has previously been explored in other alpha-synuclein-related PD models^17^. For example, the alpha-synuclein mutants A53T and A30P bind to the lysosomal receptor LAMP2A more tightly than wild-type alpha-synuclein, which prevents their degradation and cargo loading of other chaperone-mediated autophagy substrates into lysosomes^17^. Chaperone-mediated autophagy impairment and alpha-synuclein accumulation could explain the neuronal degeneration in our rat model. This neurodegeneration is accompanied by a strong neuroinflammation, which may enhance neuronal dysfunction and degeneration.

In contrast, Tubastatin A treatment increases the protein expression levels of Hsc70 and Lamp2A, and reduces the expression levels of human alpha-synuclein, and pS129 alpha-synuclein, possibly as a consequence of increased chaperone-mediated autophagy activity. Tubastatin A may also result in increased alpha-synuclein acetylation, thereby increasing its recognition by degradation pathways. In addition, astrogliosis was reduced in Tubastatin A-treated animals. This could be a consequence of reduced dopaminergic neuron loss.

However, our rat model produced a mild neurodegeneration of the dopaminergic neurons after injecting alpha-synuclein in the substantia nigra. We did not observe an impact of the human alpha-synuclein overexpression on the integrity of dopaminergic projections to the striatum. Therefore, this rat model is not acute enough to produce a degeneration of the dopaminergic projections to the striatum two weeks post-injection.

Other degradation pathways are also known to be involved in the degradation of alpha-synuclein. However, as we did not detect an accumulation of proteinase K-resistant alpha-synuclein inclusions at the early time point used in our study (data not shown), we focused on the major pathway for degradation of soluble alpha-synuclein, the chaperone-mediated autophagy. Our previous study using AAV6 to drive the expression of alpha-synuclein under the human synapsin-1 gene promoter resulted in stronger loss of nigral dopaminergic neurons after two weeks post-injection, and also in the accumulation of PK-resistant alpha-synuclein inclusions^8^. In future studies, it will be interesting to assess the effect of HDAC6 inhibition in the modulation of other degradation pathways, such as macro-autophagy, in models of PD developing alpha-synuclein aggregates, such as the alpha-synuclein BAC transgenic rats^27^. This model recapitulates some of the changes seen in early and late PD stages, such as hyposmia prior to the onset of locomotor defects, C-terminal truncation and conversion into insoluble alpha-synuclein species that are enriched in the nigrostriatal system, and dopaminergic neuron loss^27^.

Importantly, we found that Tubastatin A had a very prominent effect on alpha-synuclein phosphorylation at serine position 129. This toxic form of alpha-synuclein is a hallmark of alpha-synucleinopathies^21^. We recently suggested that the modulation of alpha-synuclein acetylation in lysines 6 and 10 might modify phosphorylation at serine position 129^8^. This interplay between lysine acetylation and serine phosphorylation has not been investigated in detail yet, due to the inexistence of specific acetylated-alpha-synuclein antibodies. This interplay has already been reported for other proteins, such as Tau, where acetylation of Tau in lysine 321 inhibits phosphorylation in serine 324, thereby regulating its polymerization and function^28^. Our proteome analysis did not reveal changes in the levels of known kinases or phosphatases interacting with alpha-synuclein, but it would be interesting to explore, in future studies, the cross-talk between different alpha-synuclein post-translational modifications, and how they impact on alpha-synuclein function, toxicity, aggregation and degradation.

We also detected a truncated form of C-terminal alpha-synuclein around 14 kDa. Truncated forms of alpha-synuclein are found in Lewy Bodies^21^, and the C-terminal truncated alpha-synuclein, containing the S129 region, has been associated to increased toxicity in the rat model overexpressing full-length alpha-synuclein in the substantia nigra^22^. This post-translation modification would be important to validate, as it would permit to better understand if a C-terminal truncated alpha-synuclein could lead to more toxicity of the full length alpha-synuclein in our rat model. If this is confirmed, it would be interesting to study how Tubastatin A is regulating this modification.

There is a real need to discover new therapeutic targets and tools for PD and other alpha-synucleinopathies, as disease-modifying therapies are not available yet. Single-target approaches have been unsuccessful in the treatment of PD, which is a complex neurodegenerative disease combining genetic and environmental risk factors. Our study demonstrated that Tubastatin A treatment protected dopaminergic neurons within the substantia nigra of rats through the reduction of alpha-synuclein expression, up-regulation of key members of the chaperone-mediated autophagy, reduction of neuroinflammation, increase of protein acetylation, modulation of phosphorylation of alpha-synuclein, and rescue of PD-related pathological pathways. Therefore, inhibition of HDAC6 by a specific inhibitor such as Tubastatin A seems to constitute an excellent way to balancing out side effects and multi-targeting of proteins and pathways.

## Material and Methods

### Recombinant viruses

Recombinant adeno-associated virus (AAV) vectors were prepared as described^15,29^ and according to standard protocols. Serotype 2 vectors were propagated in HEK293 cells, purified by iodixanol step gradient ultracentrifugation and heparin affinity FPLC, followed by extensive dialysis against PBS. Genome copies were determined by quantitative real time PCR and purity > 99% by SDS gel electrophoresis and silver staining.

### Surgical procedure and tubastatin A treatment

All experimental animal procedures were conducted according to approved experimental animal licenses, issued by the responsible animal welfare authority (Niedersaechsisches Landesamt für Verbraucherschutz und Lebensmittelsicherheit) and controlled by the local animal welfare committee and veterinarians at the University Medical Center Goettingen after approval from the ethic committee (license number 15/1799). During animal experiments, measurements were implemented to meet the requirements of replacement, refinement or reduction. Young adult female Wistar rats (230–250 g each; Janvier, Saint Berthevin, France) were used for AAV intracerebral injections. Rats were housed in a temperature-controlled room that was maintained on a 12-h light and/or dark cycle. Food and water were provided ad libitum.

All surgical procedures were performed under general anaesthesia using a mixture of 70 mg/kg Ketamin-Hydrochlorid and 10 mg/kg Xylazin. After placing the animal into a stereotaxic frame (David Kopf Instruments, USA), 2 μL of human WT alpha-synuclein AAV2 virus (~1.10^8^ vector genomes [vg]/ml) was injected unilaterally into the right substantia nigra pars compacta (SNpc) at a flow rate of 0.5 μl/min using the following stereotaxic coordinates: −4.7 mm anteroposterior, −2.2 mm mediolateral from the bregma, and −6.7 mm dorsoventral (DV) from the dura. The tooth bar was adjusted to −2.3 mm. Injection was performed using a stainless canula attached to a 10 μL Hamilton syringe with a 22 s gauge needle. After delivery of the viral vector, the canula was retracted 0.1 mm DV, held in place for 5 min and slowly withdrawn from the brain.

Tubastatin A is a very selective HDAC6 inhibitor with an IC50 of 4 nM and 100 to over 1000-fold selectivity for HDAC6 over other HDAC classes^30^.

For Tubastatin A treatment, Tubastatin A HCl powder (500 mg, InVivoChem, V0281) was dissolved in DMSO and diluted in PBS supplemented with 2% Tween 20 before use in animals. Rats were given daily 15 mg/kg Tubastatin A by intraperitoneal injection, two days after virus intracerebral injection until sacrifice. Animals were sacrificed 2 weeks after virus intracerebral injection for histological and biochemical analysis.

### Histological studies and image analysis

#### Tissue preparation

Rats were transcardially perfused with 200 ml PBS until clear flow, then with 4% PFA in PBS at a flow rate of 10 ml/min. Brains were removed, post-fixed overnight in 4% PFA solution, then cryoprotected by immersion in 30% sucrose for 36 hours. Cryoprotected brains were embedded in Jung Tissue Freezing Medium (Leica Microsystems) and cut on a cryostat (Leica CM3050S) at a 30 µm thickness. Sections were collected as serial coronal free-floating sections in a storage solution (0.1 M PBS; 0.1% sodium azide), stored at 4 °C until use.

### Immunofluorescence

The sections were blocked by incubation with 4.5% normal goat serum (NGS) in PBS for one hour, then incubated overnight at 4 °C in PBS supplemented with 3% NGS, 0.2% Triton X-100 and one of the following antibodies: anti-alpha-synuclein (1:500; mouse; (211) Santa-Cruz), anti-phospho S129-alpha-synuclein (1:500; rabbit; EP1536Y, Abcam), anti-TH (1:1000; rabbit; Millipore), anti-HSC70 (1:500; mouse; B-6, Santa-Cruz), anti-LAMP2A (1:500; rabbit; EPR4207(2), Abcam), anti-GFAP (1:500; rabbit; Millipore), anti-S100β (1:100; rabbit; EP1576Y, Abcam), anti Iba1 (1:1000; rabbit; EPR16588), Abcam), anti-acetylated lysines (1:100; rabbit; Millipore). Sections were rinsed three times in PBS and incubated with Alexa-Fluor 488-labeled anti-mouse IgG or Alexa Fluor 594-labeled anti-rabbit IgG (Life Technology) for 1 h at room temperature. The sections were rinsed in PBS and the nuclei were counterstained with DAPI (dilution 1:10,000, Wako) for 3 minutes. The sections were mounted on glass slides and coverslips in Aqua-Poly/Mount (Polysciences), and stored at 4 °C in the dark until further processing. Images of the immunostained sections were taken within 48 h.

#### Stereology

The total number of dopaminergic neurons was determined by counting the number of TH-immunoreactive cells by stereology in every fourth brain section in the region of the SNpc (7 sections throughout the substantia nigra). Ventral tegmental TH-positive cells were discarded. StereoInvestigator software (MicroBrightField, Bioscience) was used to count cells in an unbiased manner. The estimation of the total number of TH-positive neurons per SNpc was achieved using the optical fractionator method^31^.

### Microscopy

Images were acquired using a fluorescence microscope Leica DMI4000B, or a laser-scanning confocal immunofluorescent microscope C2 Plus (Nikon) from the Nikon Imaging Center, Heidelberg, Germany.

### Biochemical studies

#### Tissue processing for biochemical studies

After sacrifice, rat brains were rapidly collected and sliced into 1 mm-thick coronal sections with a brain matrix. The midbrain was dissected out and fast-frozen in liquid nitrogen before storage at −80 °C until use.

#### Fractionation experiments

For fractionation experiments, samples were processed as previously described^32^. Briefly, frozen pieces of tissue were weighted and placed in a loose-fitting Dounce homogenizer with 25 volumes of ice-cold Buffer A [10 mM HEPES pH 7.9, 10 mM KCl, 0.1 mM EDTA, 0.1 mM EGTA, 1 mM DTT, 1X Halt protease and phosphatase inhibitor cocktail (Thermo Scientific)] and homogenized with 10 up-and-down passes. We added 0.5% Nonidet P-40 and the mixture was fully homogenized by 15 up-and-down passes. The homogenate was transferred to an Eppendorf tube and incubated on ice for 10 minutes. We used the homogenate as the total fraction. The rest was centrifuged at 11,000 g for 7 minutes at 4 °C. The supernatant was retained as the cytosolic fraction. The pellet was homogeneously resuspended in 500 μL of Buffer A and centrifuged at 11,000 g for 7 minutes at 4 °C. The supernatant was discarded.

The pellet was resuspended in 1/10 of initial volume of Buffer B [20 mM HEPES pH 7.9, 400 mM NaCl, 1 mM EDTA, 1 mM EGTA, 1 mM DTT, 1X Halt protease and phosphatase inhibitor cocktail (Thermo Scientific)]. This fraction was sonicated for 20 seconds at 20% amplitude, vortexed at five-minute intervals over a 15-minute period and centrifuged at 11,000 g for 5 min at 4 °C. The supernatant constituted the nuclear fraction. Protein concentration was determined by Bradford protein assay (Thermo Scientific).

#### Western blotting

Protein samples extracted from tissue were diluted in NuPAGE LDS sample buffer (Thermo Fisher Scientific) with 1 M DTT, boiled at 70 °C for 10 min, loaded on a 4–12% Bis-Tris gel (Thermo Fisher Scientific), and subjected to SDS-PAGE on MES buffer (Thermo Fisher Scientific) for 35 min. PageRuler prestained protein ladder (Thermo Fisher Scientific, USA) was also loaded on the gel. Gels were either stained using colloidal Coomassie brilliant blue G-250, or proteins from the gel were transferred to a PVDF membrane on a wet transfer system in NuPAGE transfer buffer (Life Technologies). Proteins on the membrane were fixed by incubation of the membrane in 0.4% PFA-PBS for 1 h at RT. The membrane was then blocked in Odyssey blocking buffer (Li-Cor) and Tris-buffered saline (TBS) (ratio 1:1) for 1 h at RT before incubation overnight at 4 °C with one of the following antibodies: anti-alpha-synuclein (1:500; mouse; (211) Santa-Cruz), anti-phospho S129-alpha-synuclein (1:500; rabbit; EP1536Y, Abcam), anti-TH (1:2000; rabbit; Millipore), anti-HSC70 (1:1000; mouse; B-6, Santa-Cruz), anti-LAMP2A (1:1000; rabbit; EPR4207(2), Abcam), anti-total alpha-tubulin (1:5000; rabbit; 11H10, Cell Signaling Technology), anti-acetylated alpha-tubulin (1:500; mouse; 6-11B-1, Sigma), anti-GAPDH (1:5000; rabbit; Abcam), anti-acetylated lysines (1:1000; rabbit; Millipore) diluted in Odyssey blocking buffer and TBS supplemented with 0.1% Tween 20. The membrane was rinsed and incubated with Li-Cor secondary antibodies for 1 h at RT (1:10,000, Li-Cor, IRDye 800CW donkey anti-mouse or IRDye 680CW donkey anti-rabbit). Antibody binding was detected with the Odyssey CLx imaging system (Li-Cor). Bands were quantified with LI-COR Image Studio 3.0 software.

#### Mass spectrometry

Proteins were separated by SDS-PAGE on a 4–12% Bis-Tris Plus gel, 10-well, 1 mm thick (ThermoFisher). Electrophoresis was stopped when the proteins had entered 3 cm of the running gel. After Coomassie blue staining, each lane was cut into four pieces.

In-gel reduction, alkylation, digestion. The proteins were reduced with 60 µL 40 mM dithiothreitol (DTT; Sigma-Aldrich, Taufkirchen, Germany) in 50 mM TEAB, pH 8.5 at 57 °C for 30 min and alkylated with 60 µL 50 mM iodoacetamide (IAA; Sigma-Aldrich, Taufkirchen, Germany) in 50 mM TEAB, pH 8.5 at 25 °C for 20 min in the dark. Gel pieces were dehydrated with 60 µL 100% ACN and washed with 60 µL 50 mM TEAB, pH 8.5. A total of 30 µL of 8 ng/µL in 50 mM TEAB trypsin solution (sequencing grade, Thermo-Fisher, Rockford, USA) was added to the dry gel pieces and incubated for 6 h at 37 °C. The reaction was quenched by addition of 20 µL of 0.1% trifluoroacetic acid (TFA; Biosolve, Valkenswaard, The Netherlands). The resulting peptides were extracted by dehydrating twice for 20 min each in 20 µL ACN and washing in 30 µL 50 mM TEAB, pH 8.5. The supernatant from each extraction step was collected and dried in a vacuum concentrator before LC-MS analysis. Samples were diluted in 15 ul 0,1% TFA, 99,9% water.

LC-MS/MS measurements. Nanoflow LC-MS^2^ analysis was performed with an Ultimate 3000 liquid chromatography system directly coupled to an Orbitrap QE HF mass spectrometer (both Thermo-Fischer, Bremen, Germany). Samples were delivered to an in-house packed analytical column (inner diameter 75 µm × 20 cm; CS – Chromatographie Service GmbH, Langerwehe, Germany) filled with 1.9 µm ReprosilPur-AQ 120 C18 material (Dr. Maisch, Ammerbuch-Entringen, Germany). Solvent A was 0.1% formic acid (FA; ProteoChem, Denver, CO, USA) and 1% ACN (Biosolve) in H_2_O (Bisolve). Solvent B was composed of 0.1% FA (ProteoChem), 10% H_2_O (Biosolve) and 89.9% ACN (Biosolve). The sample was loaded to the analytical column for 20 min with 3% B at 550 nL/min flow rate. The peptide separation was carried out with 25 min linear gradient (3–40% B) with the reduced flow rate of 300 nL/min. The mass spectrometer was operated in data-dependent acquisition mode, automatically switching between MS and MS^2^. MS spectra (m/z 400–1600) were acquired in the Orbitrap at 60,000 (m/z 400) resolution. Fragmentation in HCD cells was performed for up to 15 precursors MS^2^ spectra were acquired at 15,000 (m/z 400) resolution.

Database search. Raw files were processed using MaxQuant (version 1.5.3.30^33^) for peptide identification and quantification. MS^2^ spectra were searched against the uniprot-Rattus-norvegicus-UP000002494 database (containing 7989 sequences) and uniprot-Rattus-norvegicus-UP000002494-additional database (containing 21985 sequences) (both retrieved in July, 2017) contaminants of Maxquant were included. We defined carbamidomethylation of cysteine residues as fixed modification and acetyl (protein N-term), oxidation of methionine, deamidation of asparagine and glutamine acid as variable modifications and trypsin/P as the proteolytic enzyme with up to 2 missed cleavages allowed. The maximum false discovery rate for proteins and peptides was 0.01 and a minimum peptide length of 7 amino acids was required. Analysis was done in LFQ mode. All other parameters are default parameters of MaxQuant.

Perseus data analysis. The MaxQuant file was analyzed using Perseus software (version 1.6.2.1^34^). LFQ intensity of three Veh-treated rats and four Tubastatin A-treated rats was processed. After log 2 transformation, filter based on a minimum of 3 valid values in at least one group was applied and significantly differently expressed proteins were identified by using a two-sample t-test. The GO terms enrichment was calculated with Fisher’s exact test implemented in the Perseus environment and compared to the DAVID bioinformatics database for functional annotations (https://david.ncifcrf.gov).

Protein network analysis. String 10.0 bioinformatics software (https://string-db.org) was used to search for and represent protein-protein interactions between the proteins involved in the GO terms found in the up- and down-regulated clusters in the Tubastatin A-treated rats. The network edges were based on evidence. Textmining was excluded from the active interaction sources. Medium confidence (0.400) was applied for interaction score.

### Statistical analysis

Statistical analyses were conducted using GraphPad Prism 5 software. All data are presented as means ± standard error of the mean (SEM). Unpaired Student’s *t* test was applied for individual pairwise comparisons in all cases. For multiple comparison tests of more than two groups and study of two factors (injection of alpha-synuclein VS non-injection, and treatment vehicle VS Tubastatin A), two-way ANOVA analyses were followed by a Bonferroni *post hoc* test. The threshold for statistical significance was P < 0.05.

## Data Availability

Data supporting the findings of this study are available within the article and from the corresponding author upon reasonable request.
